# The Effects of Warfarin and Direct Oral Anticoagulants on Systemic Vascular Calcification: A Review

**DOI:** 10.3390/cells10040773

**Published:** 2021-03-31

**Authors:** Kalaimani Elango, Awad Javaid, Banveet K. Khetarpal, Sathishkumar Ramalingam, Krishna Prasad Kolandaivel, Kulothungan Gunasekaran, Chowdhury Ahsan

**Affiliations:** 1 Department of Cardiology, University of Nevada Las Vegas School of Medicine, 1701 W Charleston Boulevard Ste. 250, Las Vegas, NV 89102, USA; kalaimani.elango@gmail.com (K.E.); chahsan92@yahoo.com (C.A.); 2 Department of Internal Medicine, University of Nevada Las Vegas School of Medicine, 1701 W Charleston Boulevard Ste. 250, Las Vegas, NV 89102, USA; awadiqbaljavaid@gmail.com (A.J.); khetarpal.banveet@gmail.com (B.K.K.); 3 Division of Hospital Medicine, Lovelace Medical Center, 601 Dr. Martin Luther King Jr. Avenue NE, Albuquerque, NM 87102, USA; sathishmed@gmail.com; 4 Division of Oncology, St. Joseph Medical Center, 523 N 3rd Street, Brainerd, MN 56401, USA; prasadarsenal87@gmail.com; 5 Division of Pulmonary Diseases and Critical Care, Yale New Haven Health Bridgeport Hospital, 267 Grant Street, Bridgeport, CT 06610, USA

**Keywords:** warfarin, direct oral anticoagulants, vascular calcification, valvular calcification, vitamin K, matrix gla protein

## Abstract

Warfarin has been utilized for decades as an effective anticoagulant in patients with a history of strong risk factors for venous thromboembolism (VTE). Established adverse effects include bleeding, skin necrosis, teratogenicity during pregnancy, cholesterol embolization, and nephropathy. One of the lesser-known long-term side effects of warfarin is an increase in systemic arterial calcification. This is significant due to the association between vascular calcification and cardiovascular morbidity and mortality. Direct oral anticoagulants (DOACs) have gained prominence in recent years, as they require less frequent monitoring and have a superior side effect profile to warfarin, specifically in relation to major bleeding. The cost and lack of data for DOACs in some disease processes have precluded universal use. Within the last four years, retrospective cohort studies, observational studies, and randomized trials have shown, through different imaging modalities, that multiple DOACs are associated with slower progression of vascular calcification than warfarin. This review highlights the pathophysiology and mechanisms behind vascular calcification due to warfarin and compares the effect of warfarin and DOACs on systemic vasculature.

## 1. Introduction

Anticoagulants are an effective tool to reduce morbidity and mortality in patients suffering from arterial thromboembolism and venous thromboembolism. As the global population continues to increase in average age and the burden of cardiovascular disease (CVD) increases, the challenge of reducing this burden remains as difficult as ever [1]. In the setting of increasing CVD, the demand for oral anticoagulants is as high as it has ever been. Millions of patients are on lifelong anticoagulant therapy for various indications. Since the 1960s, warfarin has been used effectively to prevent thromboembolism. Because of its drawbacks, effort was put forth to find a more convenient drug for patients. During the last 10 years, direct oral anticoagulants (DOACs) have gained prominence due to their preferable side-effect profile and ease of use. Due to the cost and relatively recent approval of DOACs, warfarin is more affordable and still has a broader range of indications. Worldwide, prescription trends are now showing that warfarin use continues to decline, while DOAC use is on the rise [2,3]. One of the known but uncommonly highlighted clinical aspects of warfarin’s side-effect profile is its effect on vascular calcification. Numerous studies have shown that warfarin has a higher burden of calcification when compared to controls [4]. This review analyzes the mechanisms which promote calcification in warfarin users and examines the effects of DOACs and warfarin on systemic vasculature.

## 2. Warfarin

### 2.1. History

In 1921, cattle in North Dakota and Canada began dying mysteriously after suffering from severe internal bleeding. Farmers had switched their cattle fodder from corn to sweet clover hay species *Melilotus alba* and *Melilotus officinalis* [5]. If these species are stored in damp conditions, they are prone to infection by molds. The spoiling process can convert the plants’ natural coumarins into dicumarol, which has anticoagulant properties [6]. Veterinarians Frank Schofield and Lee Roderick determined that cattle consuming spoiled sweet clover were affected by the disease [7]. In the 1930s, Karl Paul Link discovered that the culprit molecule was dicoumarol, and in the 1940s, warfarin began to be used as rodenticide [8]. Warfarin was introduced as an anticoagulant in the 1940s and after further testing, was approved for use as an anticoagulant in the United States in 1954 [5]. Notably, President Dwight Eisenhower was treated for a myocardial infarction (MI) with warfarin in 1955, after which it began to become a household name and started to be more widely used in the 1960s.

### 2.2. Mechanism of Action and Properties

**Figure 1:**
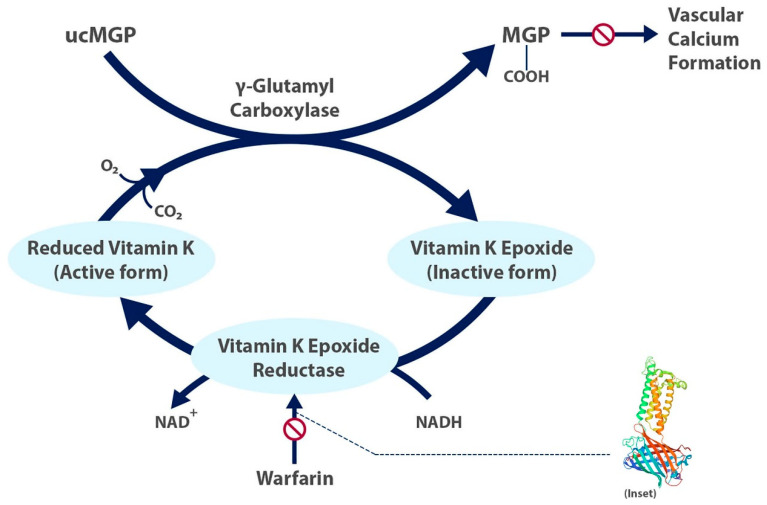
The carboxylated, active form of matrix gla protein prevents vascular calcium formation and relies on the active form of vitamin K. Warfarin inhibits formation of the active form of vitamin K. Inset shows structure of human vitamin K epoxide reductase with warfarin [12]. ucMGP = uncarboxylated matrix gla protein.

**Figure 2:**
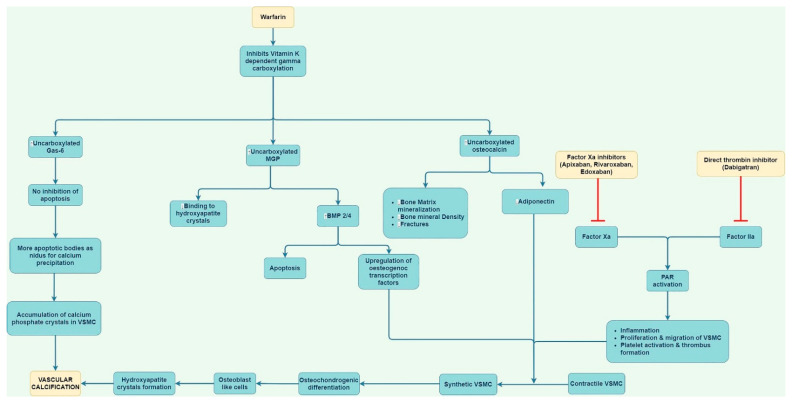
Summary figure showing mechanism and implication of warfarin and direct oral anticoagulants (DOACs) on vascular calcification. Warfarin causes calcification due to inhibition of vitamin-K-dependent carboxylation. DOACs may prevent calcification by inhibiting protease-activated receptors (PAR) activation. Gas-6 = growth arrest specific 6 protein; VSMC = vascular smooth muscle cells; MGP = matrix gla protein; BMP = bone morphogenetic protein.

Factors II, VII, IX, and X and proteins C and S are blood clotting factors synthesized as inactive precursors. They undergo a vitamin-K-dependent post-translational modification, which involves the gamma carboxylation of glutamic acid residues [9]. These gamma-carboxyglutamyl residues bind calcium ions and are necessary for interaction with platelets. The complex of clotting factors with calcium ions binds phospholipids on platelets. The reaction is catalyzed by gamma-glutamyl carboxylase and requires carbon dioxide, oxygen, and the reduced form of vitamin K [9]. The reduced vitamin K cofactor is converted to vitamin K epoxide during the reaction. Vitamin K is regenerated from the epoxide by vitamin K epoxide reductase (Figure 1). Warfarin acts by inhibiting vitamin K epoxide reductase (Figure 2). The four coagulation factors involved have differing half-lives, up to 60 h [9].

Factor VII has a half-life of 6 h, and therefore, warfarin begins to have an effect within approximately 24 h. Consequently, reversal following administration of vitamin K takes approximately 24 h. The peak effect may take up to 96 h. Protein C is a vitamin-K-dependent serine protease activated by thrombin. The activated form uses protein S as a cofactor and degrades factors Va and VIIIa. Both protein C and S are anticoagulant factors, and for this reason, a bridging agent, usually heparin, is necessary during a temporary hypercoagulable state that occurs before warfarin begins to take effect. Warfarin has no direct effect on an established thrombus, but once a thrombus has occurred, the goal of treatment is to prevent further enlargement of the formed clot. The anticoagulant effects of warfarin can be overcome by the administration of vitamin K.

The chemical properties of warfarin can influence metabolism and dosing in clinical practice. Warfarin is administered in the form of a racemic mixture of R and S enantiomers. The S-enantiomer is more active and possesses 2–5 times greater potency than the R-enantiomer [10,11]. Each isomer is metabolized distinctly in the liver. Oxidative metabolism of the S- isomer is affected by CYP2C9 [10]. The two most common genetic polymporphisms of CYP2C9 are CYP2C9*2 and CYP2C9*3 [11]. Multiple studies have confirmed that patients with these enzyme variants have reduced enzyme activity and require lower doses of warfarin to maintain a therapeutic INR [11].

## 3. Direct Oral Anticoagulants

### 3.1. History

Over the last 10 years, options for oral anticoagulation have greatly expanded with the advent of DOACs. These drugs are small molecules that are renally cleared and work by inhibiting thrombin or factor Xa. In 1884, John Berry Haycraft discovered that medicinal leeches have anticoagulant properties [13]. Hundreds of years later, with new biochemical techniques, recombinant hirudin was shown to prevent thromboembolism in the 1990s. Eventually, direct thrombin inhibitors were found to be effective in preventing thrombosis. Bivalirudin was approved in the United States in 2000, and dabigatran was approved in the European Union in 2008 and the United States in 2010. Factor Xa inhibitors were also discovered by way of leeches. Antistasin, the 15 kilodalton peptide isolated from the salivary glands of the Mexican leech *H. officinalis* paved the way for further research after it was found to block factor Xa activity [14]. Subsequently, tick anticoagulant peptide was discovered to do the same, and eventually rivaroxaban was the first factor Xa inhibitor to be approved in 2008.

### 3.2. Mechanism of Action and Properties

DOACs are divided into two main groups based on their mechanisms of action: direct thrombin inhibitors and factor Xa inhibitors. Thrombin has multiple significant roles in hemostasis, including the conversion of factor V to Va, the conversion of fibrinogen to form fibrin, and the activation of factor XIII, the activation of platelets, and the endothelial release of prostacyclin and tissue plasminogen activator [9]. In this manner, direct thrombin inhibitors are effective because they affect many different pathways. Factor Xa inhibitors bind the active site of factor X. Factor X represents an important stage in the coagulation cascade, where the intrinsic and extrinsic pathways meet. Thus, inhibiting factor X prevents generation of thrombin from both pathways [15]. Because both direct thrombin inhibitors and factor Xa inhibitors have predictable pharmacokinetics, routine monitoring is not necessary.

## 4. Advantages and Disadvantages of Warfarin and Direct Oral Anticoagulants

Warfarin has many benefits in comparison to DOACs. Warfarin is considerably more affordable, especially for patients who are uninsured. It is approved to treat a wider range of conditions, due to the numerous clinical trials that have studied its effects. It also has a relatively straightforward reversal process, whereas DOACs have expensive reversal agents. On the other hand, studies have shown that patients’ international normalized ratio (INR) is not within goal range for a significant amount of time [16]. Patients require frequent blood draws and INR checks, which can become cumbersome and can lead to lack of compliance. In addition, bridging is required in patients taking warfarin, and peak effect may take up to 96 h. The major advantage of DOACs is the lack of need to monitor INR and to frequently adjust doses. There are less drug and diet interactions than warfarin, and the occurrence of significant bleeding has been found to be similar or better. DOACs can also be used in patients who have genetic warfarin resistance. DOACs also have a faster time to peak than warfarin and a shorter half-life, which is advantageous when anticoagulation must be held [9]. Although warfarin has a broader range of indications, this advantage continues to abate, as DOACs continue to be studied. For example, recent studies have shown that DOACs are an effective anticoagulant for patients diagnosed with atrial fibrillation and atrial thrombus [17]. Disadvantages include expensive reversal agents, increased rate of adverse events in patients with chronic kidney disease (CKD) and liver disease, and a less broad range of indications than warfarin.

## 5. Vascular Calcification

Vascular calcification has been shown in multiple studies to correlate with MACE (major adverse cardiovascular events) [18,19,20,21]. Calcification has been implicated in cerebrovascular accidents, coronary artery disease, peripheral artery disease, and CKD [22,23,24,25]. Thus, researchers and clinicians have sought methods to reduce calcium burden in patients.

### 5.1. Pathophysiology of Vascular Calcification

Vascular calcification is defined as the pathologic deposition of calcium-phosphate in the systemic vasculature and can occur in the arterial intima as well as the media. The pathogenesis of vascular calcification is complex and involves inflammation, autophagy defects, endoplasmic reticulum stress, and mitochondrial dysfunction [23]. Traditional risk factors for vascular calcification include hypertension, diabetes, hyperlipidemia, age, genetics, and smoking. The first step in calcification is likely the conversion of smooth muscle cells into osteoblast-like cells. Increased secretion of matrix proteins then occurs, osteoblast-like cells mineralize, and then calcium and phosphorus are deposited [26]. Intimal calcification was previously thought to occur late in the disease course; however, intravascular ultrasound (IVUS) has shown that intimal calcification can also occur early in disease [26]. Medial calcification occurs secondary to inflammation, as well as conversion of smooth muscle cells into osteoblastic cells. Elevated serum levels of calcium and phosphate result in the deposition of hydroxyapatite in the media. Intimal calcification over time contributes to atherosclerosis, stenosis of vessels, and ischemic events such as MI. Medial calcification can lead to stiffening and decreased compliance of arteries, which itself is associated with cardiovascular mortality [27]. Vascular calcification should be differentiated from atherosclerotic calcification. In vascular calcification, calcium deposits directly into the vessel intima or media. In atherosclerotic calcification, chronic endothelial injury in the setting of various insults, causes endothelial dysfunction, macrophage activation, smooth muscle proliferation, and proliferation of macrophages which consume smooth muscle cells and lipids. Eventually an atheroma forms, which progresses to an advanced lesion, in which calcium is deposited [28]. Valvular calcification can occur secondary to age-related degenerative processes and is most clinically relevant in the aortic valve, where it can lead to aortic stenosis. Vascular calcification is an important step in the process of atherosclerosis. Although some studies show that calcified plaque is more stable than noncalcified plaque, calcified plaque is a predictor of cardiovascular events and recurrent stroke [29].

### 5.2. The Role of Vitamin K

As per Virchow’s Triad, endothelial injury, stasis or turbulent blood flow, and hypercoagulability of the blood combine to cause thrombosis [28]. After endothelial injury occurs, coagulation acts as a protective mechanism to prevent bleeding. Oral anticoagulants work at multiple different sites of the coagulation cascade to prevent thrombosis. Warfarin acts by inhibiting activation of factors II, VII, IX, and X, whereas DOACs work by directly blocking the activity of factor Xa and thrombin. Vitamin K plays a key role in the coagulation pathway, as it is a cofactor required for the activation of several carboxylase enzymes in the liver. Coagulation factors II, VII, IX and X undergo a vitamin-K-dependent post-translational modification, whereby their glutamic acid residues are carboxylated to form gamma carboxy-glutamic acid residues [9]. Warfarin shares a common ring structure with vitamin K, which allows warfarin to be a vitamin K antagonist. Vitamin K exists in two forms: vitamin K1 and vitamin K2. Vitamin K1 is found in green vegetables, such as collard greens, broccoli, kale, and spinach. Vitamin K2 is formed by vitamin K1 in the body as well as gut bacteria and can be found in meats, eggs, cheese, and certain seafood. In the Rotterdam Study, vitamin K2 intake was associated with a decrease in all-cause mortality and severe aortic calcification after adjusting for age, gender, BMI, smoking, diabetes, education, and diet [30]. As vitamin K has clear associations with slower progression of systemic calcification, its inhibition by warfarin is central to long- term effects in the vasculature of warfarin users.

## 6. Mechanisms by Which Warfarin May Cause Calcification

### 6.1. Impact of Matrix Gla Protein

Matrix gla protein (MGP) is a mineral binding, extracellular matrix protein that has been shown to inhibit arterial calcification. MGP is secreted by chondrocytes and vascular smooth muscle cells in the arterial tunica media [31]. There is evidence that MGP is also secreted by endothelial cells. Many mechanisms have been suggested to explain the process by which MGP inhibits calcification, including vesicle-related mechanisms and by adsorbing to hydroxyapatite crystals [32,33]. The glutamate residues of MGP attract ions such as calcium, phosphate, and hydroxyapatite, which together combine to form an extracellular matrix. Studies have shown that MGP reduces the accumulation of calcium phosphate deposition in vessel walls [34]. As a result of binding calcium ions, MGP clears calcium from circulation and inhibits calcium crystal growth [35]. MGP has been found to bind multiple extracellular membrane components, such as vitronectin and elastin, which prevent the formation of calcium complexes [35].

MGP also prevents vascular smooth muscle cells from differentiating into osteogenic cells which contribute to calcification. This process is related to MGP’s interplay bone morphogenetic protein-2, which is part of a family of bone morphogenetic proteins (BMP) that play a complex role in vascular calcification. The main function of BMP is to maintain and repair bone. Expression of BMP has been found in atherosclerotic lesions, and its induction leads to oxidative stress, inflammation, and oxidized lipids [36]. BMP-2 stimulates differentiation of vascular mesenchymal cells into osteoblast-like, which can lead to bone formation in the artery wall [36]. When MGP expression is reduced, unopposed expression of BMP-2 generates vascular calcification [36].

Apoptosis is a key step in the process of calcification, and MGP has been shown to regulate this process [37]. MGP has been found in apoptotic bodies released from vascular smooth muscle cells. These bodies have shown to act as a nidus for calcium formation and have been found in atherosclerotic lesions [38]. MGP expression is highest when apoptosis is at its highest activity in vascular smooth muscle cells, which further provides evidence of the relationship [38]. Mice without MGP have been shown to have extensive, accelerated vascular calcification associated with mortality [39]. As warfarin inhibits the activity of MGP, it can cause dysregulation of apoptosis in vascular smooth muscle, which can lead to vascular smooth muscle proliferation and eventually vascular calcification and atherosclerosis.

Mice with mutated MGP have been shown to develop Keutel syndrome, an autosomal recessive disease in which patients develop excessive calcification of the cartilage and tracheal stenosis [38]. There are conflicting data on whether MGP can be employed as a marker for early clinical intervention in patients with arterial calcification [40]. In one study, the carboxylated form of MGP was found near healthy cells of arterial media, whereas the uncarboxylated form was found near atherosclerotic lesions [41]. This finding indicates that MGP must be carboxylated to have its calcium inhibiting effect. MGP depends on vitamin K, and therefore, warfarin reduces gamma-carboxylation of MGP. As MGP has such a central role in calcification, one can quickly realize why its inhibition by warfarin can eventually cause atherosclerosis.

### 6.2. Other Mechanisms

Although MGP likely plays a large role in warfarin’s effect on calcification, there are other proposed mechanisms. Warfarin inhibits carboxylation of osteocalcin, which leads to reduced binding of hydroxyapatite in the uncarboxylated form [42]. Osteocalcin is a regulator of vascular calcification through adiponectin, which inhibits the differentiation of vascular smooth muscle cells into osteoblast cells [43]. The role of osteocalcin in bone regulation provides mechanistic insight into the common pathways between vascular calcification and bone formation. Indeed, multiple studies have shown that warfarin use is associated with reduced bone mineral density (BMD). In one such analysis in 2020, among patients using warfarin more than one year compared to a control group, a longer time of warfarin use increased the likelihood of having low BMD (odds ratio (OR): 1.239, confidence interval (CI): 1.064–1.674, *p* = 0.01) [44]. The warfarin group had significantly lower bone density in all anatomical sites, including spine, hip, and femoral neck. In addition, bone gla protein, periostin, gla-rich protein, and growth-arrest specific protein 6 may all depend on vitamin K carboxylation, and their inhibition by warfarin may be linked to vascular calcification [45].

## 7. Mechanisms by Which Direct Oral Anticoagulants May Prevent Vascular Calcification

Studies on mechanisms by which DOACs may prevent vascular calcification are largely absent, as the finding that DOACs can prevent progression of vascular calcification is a relatively recent one. However, some mechanisms have been proposed as to how DOACs are involved in atherosclerosis. Thrombin is known to activate multiple protease-activated receptors (PARs), which may be responsible for inflammation and atherogenesis [46]. In endothelial cells, thrombin is involved in the production of multiple proinflammatory cytokines which may contribute to atherosclerosis and calcification. Thrombin-induced protease-activated receptor-1 (PAR-1) activation can create vascular smooth muscle migration and proliferation, which is a key step in atherosclerosis [47]. Factor Xa has similar effects on PARs and has been associated with increased inflammation, fibrosis, and vascular endothelial changes [46]. In a recent study, factor Xa was shown to be a direct agonist of PAR-1, leading to activation of platelets and formation of thrombus [48]. Studies in mice have shown that dabigatran attenuates arterial thrombosis, reduces atherosclerotic lesion size, and may lead to atherosclerotic plaque stability [49]. Additionally, the ARISTOTLE trial showed that apixaban was associated with less ischemic events than warfarin in patients with nonvalvular AF [50]. Other pathophysiologic mechanisms of reduced atherosclerosis in DOACs have been shown as well; edoxaban was shown to prevent vascular maladaptive remodeling compared to warfarin in mice [51], and rivaroxaban has been shown to improve patients’ arterial pulse wave velocity and augmentation index after switching from warfarin [52]. A summary graphic in Figure 2 displays the mechanisms behind vascular calcification with regard to warfarin and DOACs.

## 8. Studies Linking Warfarin to Calcification

Warfarin has been demonstrated in many studies to be associated with increased vascular and valvular calcification. In patients treated with warfarin, investigators found that serial IVUS showed coronary calcification progression at a faster rate than in warfarin nonusers, independent of age, statin use, and renal function [22]. In a study on warfarin’s effect on progressive coronary calcification and destabilization of atherosclerotic plaques, warfarin use was found to be associated with increased intimal and medial calcification [53]. Interestingly, at least six studies have shown that breast artery calcification has a positive association in relation to coronary artery calcification [54]. In turn, research has also shown that warfarin use is associated with increased breast calcification [55]. One example of this is a 2015 study, which compared screening mammograms from women with current, past, or future warfarin use to mammograms in women who had never used warfarin. When adjusted for age and presence of diabetes, women with history of warfarin use had 50% greater arterial calcification (39.0% versus 25.9%; *p* < 0.0001) [56]. MGP is very insoluble and therefore adheres to the vascular matrix [57]. This may be why warfarin treatment less than 6 months has not been associated with increased arterial calcification in some studies [56]. In a study published in the *Journal of the American Heart Association*, 430 patients were identified who were currently, or in the past, on warfarin therapy, and these patients had significantly more lower-extremity artery calcification than warfarin in non-users, even when controlled for age, sex, diabetes status, and creatinine [58].

## 9. Effects of Warfarin vs. DOACs on Vascular Calcification

As evidence began to mount regarding the association between warfarin and vascular calcification and DOACs became more widely available, a handful of studies examined the effects of DOACs versus warfarin in regard to vascular calcification. Recent data strongly point toward DOACs being associated with less progression of vascular and valvular calcification when compared to warfarin (Table 1). A cross-sectional study of 236 patients on rivaroxaban, dabigatran, and warfarin showed, on propensity-score-adjusted logistic regression, that warfarin use was significantly associated with calcification of the ascending aorta (OR: 2.31; 95% confidence interval (CI): 1.16–4.59; *p* < 0.017), descending aorta calcification (OR: 2.38; 95% CI: 1.22–4.67; *p* < 0.012), and a trend in aortic valve calcification (OR: 1.92; 95% CI: 0.98–3.80; *p* < 0.059) on coronary computed tomography angiography (CCTA), whereas DOAC use was not [59]. In another study of 303 patients (101 patients in each group), warfarin users had significantly greater overall coronary plaque burden, as well as a significantly higher prevalence of high-risk plaque than DOAC users and the control group [60]. Patients in the DOAC group showed no significant differences when compared to the control group. A retrospective cohort study of 202 DOAC and warfarin users showed that rivaroxaban and dabigatran had less overall plaque burden than warfarin users with a median of 2.6 years follow-up monitoring. In a randomized trial, apixaban was associated with less calcified and low attenuation plaque than warfarin on CCTA in patients with atrial fibrillation after 52 weeks of follow-up [61]. This finding persisted after adjustment for several clinical variables, including hypertension, diabetes, smoking, family history, history of percutaneous coronary intervention, coronary artery bypass graft, and aspirin and statin use. One of the largest studies was a retrospective cohort of 1748 warfarin users and 1148 DOAC users who had CT scans from 2007–2017. For each year of warfarin treatment, the odds of being in a higher coronary artery calcium (CAC) category increased (OR = 1.032, 95% CI 1.009–1.057), while DOAC treatment duration was not associated with CAC category (OR = 1.002, 95% CI 0.935–1.074) [62].

A randomized trial which studied 46 patients on rivaroxaban versus 51 patients on warfarin showed that changes in patients’ total coronary plaque volume (β 45.33, 95% CI 9.15−81.52, *p* = 0.015) and calcified plaque volume (β 24.25, 95% CI 2.95−45.55, *p* = 0.026) were significantly greater in the warfarin group compared to the rivaroxaban group [63]. The finding of greater calcification in warfarin users has not been limited to aortic and coronary vasculature; a larger burden of intracranial calcification on computed tomography assessed by experienced neuroradiologists was found in patients using warfarin compared to DOACs (β = 1.54, *p* = 0.049) [64]. Interestingly, in one randomized study, 132 hemodialysis patients were randomized to warfarin, rivaroxaban, or rivaroxaban plus vitamin K2 three times per week, over a period of eighteen months [65]. Changes in coronary artery, thoracic aorta, and cardiac valve calcium scores were not significantly different among the three study arms. However, initiation or continuation of warfarin increased levels of dephosphorylated uncarboxylated MGP, whereas levels decreased in the rivaroxaban group and rivaroxaban plus vitamin K2 group.

## 10. Effects of Warfarin and Direct Oral Anticoagulants on Valvular Calcification

In high-income countries, aortic valve stenosis is most commonly due to calcification of the aortic valve. The underlying mechanisms of aortic valve calcification in warfarin users is similar to the mechanism in vascular calcification, namely inhibition of vitamin-K-dependent proteins; however, mechanical shear stress plays a larger role in calcific aortic stenosis. Due to various molecular pathways, initiated by the infiltration of lipids and oxidative stress, with activation of inflammatory cells, the valve becomes fibrosed [66]. Even the initial stage of calcific valve disease is associated with an increased risk of MI and cardiovascular and all-cause mortality [66]. Eventually, with time, calcification of the valve leads to narrowing, left ventricular outflow obstruction occurs, and left ventricular failure can occur. As DOACs have no interaction with vitamin K, they presumably would not increase the rate of vascular calcification in patients. This is illustrated by the fact that patients with a bicuspid aortic valve almost always develop aortic stenosis. In a study of 303 patients with mild, moderate, or severe aortic stenosis, warfarin use when compared to DOAC use was associated with greater annual increases in peak aortic jet velocity and aortic valve calcification on CT, even when controlled for age, sex, hypertension, diabetes, smoking, creatinine clearance, peripheral vascular disease, and statin use [67]. Additionally, rivaroxaban and dabigatran were found in laboratory conditions to possibly inhibit the effects of factor Xa and thrombin on stenotic aortic valves. A 2018 study found that, in a population of CKD patients, rivaroxaban was associated with a reduction of both aortic and mitral valve calcification deposition and progression when compared to warfarin [68].

## 11. Considerations in Patients with Kidney Disease

Patients with CKD or end-stage renal disease (ESRD) represent a unique population. DOACs are renally excreted and require adjustment for renal function. Many patients with CKD and ESRD are unable to take advantage of the many benefits of DOACs previously outlined. Patients with CKD have a baseline elevated net calcium-phosphate balance, which can lead to vascular calcification [69]. Several studies have shown that patients with CKD and end-stage renal disease ESRD have lower vitamin K intake [70]. Balancing vitamin K intake is challenging for patients using warfarin, as they are often advised by clinicians to limit vitamin K intake to maintain goal INR. Patients with reduced renal function have long been known to have disrupted calcium and phosphate metabolism. In vitro experiments have shown that high phosphate levels in vascular smooth muscle cells increase gene expression of osteogenic factors which promote calcification [34]. In this manner, patients suffering from CKD have accelerated vascular calcification due to high baseline phosphate levels compounded by a low vitamin K diet and the indirect effect of warfarin. Although one may question whether increased vascular calcification in patients using warfarin may be confounded by CKD, several studies have controlled for this risk factor in their analyses.

Vitamin D levels tend to be low in patients with CKD and ESRD, and this deficiency has been associated with morbidity. The role of vitamin D in vascular calcification is complex; however, the bottom line appears to be that there is a biphasic relationship wherein low vitamin D levels and excess vitamin D levels have been associated with vascular calcification [71]. Large, randomized trials may be necessary to come to a final consensus.

Recent studies have shown that DOACs are relatively safe in patients with CKD and ESRD. One study of more than 20,000 patients showed that DOAC use was associated with lower risk of mortality requiring hospitalization when compared to warfarin use across all levels of renal function (hazard ratio (HR): 0.76 with *p* value < 0.001 in patients with estimated glomerular filtration rate (eGFR) > 60, HR 0.74 with *p* value < 0.001 in patients with eGFR > 30 to 60, and HR 0.76 with *p* value < 0.001 in patients with eGFR ≤ 30 or on dialysis) [72]. Bleeding requiring hospitalization was also significantly lower across all levels of renal function. Similarly, a meta-analysis earlier this year showed DOACs were associated with better efficacy in early CKD and similar efficacy and safety outcomes to warfarin in patients with CKD stages 4–5 or on dialysis [73]. In comparison to warfarin, DOACs significantly reduced the risk of stroke and VTE by 22% (HR 0.78, 95% CI 0.64–0.95) and major bleeding by 17% (HR 0.83, 95% CI 0.71–0.97). Further randomized controlled trials will be necessary to confirm these findings. As evidence continues to accumulate regarding the superiority of DOACs to warfarin in terms of vascular calcification and safety profile, DOACS may be more widely adopted in patients with CKD and ESRD.

Finally, warfarin use has been associated with the development of calciphylaxis in CKD patients in several studies [74], while this association has not been found DOAC users. In one small 2017 study, 13 of 16 patients with diagnosed calciphylaxis had stable or improved cutaneous disease after initiating either apixaban or dabigatran [75].

## 12. Conclusions

Over the last few decades, a considerable body of evidence has accumulated that warfarin leads to faster progression of vascular and valvular calcification, which may be associated with MACE. DOACs have shown in initial randomized trials that they lead to slower progression of calcification, and therefore, this may be another reason for physicians to prefer them in clinical practice. One issue to keep in mind is that DOACs have only been widely used in the last decade. Accordingly, long-term studies are necessary to determine the complete effects on systemic vasculature. All the randomized studies that were reviewed in this article have relatively short follow-up periods, and outcomes could conceivably change over a longer period. At this time, the many benefits of warfarin’s anticoagulant properties far outweigh any risks of vascular calcification. However, as DOACs continue to undergo more rigorous testing in randomized controlled trials, their superiority in terms of calcium burden may prove to be another reason warfarin continues to be less prescribed.

## Figures and Tables

**Table 1:** Effects of warfarin vs. DOACs on vascular calcification.

Author	Study Design	Study Population	Vasculature Studied	Vascular Calcification Measurement Method	Principal Findings
Hasific et al., 2020	Retrospective Cohort	2892 patients who underwent cardiac CT for various indications	Coronary arteries	CT ^1^	For each year of warfarin treatment, odds of being in higher CAC ^2^ category increased; DOAC ^3^ treatment duration was not associated with CAC category
Di Lullo et al., 2018	Retrospective Cohort	347 patients with CKD ^4^ stage 3b-4	All cardiac valves	TTE ^5^	Rivaroxaban compared to warfarin reduced both mitral and aortic valve calcifications independently of the degree of baseline calcification
Plank et al., 2018	Retrospective Cohort	303 patients with non-valvular AF ^6^	Coronary arteries	CCTA ^7^	Warfarin significantly associated with increased overall plaque and higher prevalence of high-risk plaque burden compared to DOACs and control group
Tastet et al., 2019	Retrospective Cohort	303 patients with at least mild aortic stenosis	Aortic valve	TTE and MDCT ^8^	Median annualized increase in Vpeak ^9^ larger in warfarin group compared to DOAC and no anticoagulant therapy groups
Peeters et al., 2018	Cross-Sectional	236 patients with AF	Ascending aorta, descending aorta, aortic valve	CCTA	Warfarin significantly associated with AsAc ^10^ and DAC ^11^ and trend in AVC ^12^ compared with non-anticoagulation. These same associations were absent in DOAC use when compared to non-anticoagulation
De Vriese et al., 2020	Prospective Randomized Trial	132 hemodialysis patients	Coronary arteries, thoracic aortic plaques, and cardiac valves	CT	Patients on warfarin, rivaroxaban, or rivaroxaban plus vitamin K2 did not differ significantly in terms of coronary artery, thoracic aorta, and cardiac valve calcium scores. Initiation or continuation of warfarin further increased dp-ucMGP ^13^
Lee et al., 2018	Prospective Randomized Trial	97 patients with non-valvular AF	Coronary arteries	CCTA	Warfarin significantly associated with progression of total plaque volume and calcified plaque volume when compared to rivaroxaban
Win et al., 2019	Prospective Randomized Trial	66 patients with non-valvular AF	Coronary arteries	CCTA	Significantly higher total, calcified, and low-attenuation plaque volume in warfarin group compared to apixaban

^1^ CT = computed tomography; ^2^ CAC = coronary artery calcium; ^3^ DOAC = direct oral anticoagulant; ^4^ CKD = chronic kidney disease; ^5^ TTE = transthoracic echocardiogram; ^6^ AF = atrial fibrillation; ^7^ CCTA = coronary computed tomography angiography; ^8^ MDCT = multidetector computed tomography; ^9^ Vpeak = peak aortic velocity; ^10^ AsAc = ascending aorta calcification; ^11^ DAC = descending aorta calcification; ^12^ AVC = aortic valve calcification; and ^13^ dp-ucMGP = dephosphorylated uncarboxylated MGP.
